# Organophosphate Pesticide Exposure and Neurodevelopment in Young Mexican-American Children

**DOI:** 10.1289/ehp.9828

**Published:** 2007-01-04

**Authors:** Brenda Eskenazi, Amy R. Marks, Asa Bradman, Kim Harley, Dana B. Barr, Caroline Johnson, Norma Morga, Nicholas P. Jewell

**Affiliations:** 1 Center for Children’s Environmental Health Research, School of Public Health, University of California, Berkeley, California, USA; 2 National Center for Environmental Health, Centers for Disease Control and Prevention, Atlanta, Georgia, USA; 3 Center for the Health Assessment of Mothers and Children of Salinas, Clinica de Salud del Valle de Salinas, Berkeley, California, USA

**Keywords:** Bayley Scales of Infant Development, Child Behavior Checklist, DAPs, farmworker, Mexican Americans, neurodevelopment, organophosphates, pervasive developmental disorder, pesticides

## Abstract

**Background:**

Organophosphate (OP) pesticides are widely used in agriculture and homes. Animal studies suggest that even moderate doses are neurodevelopmental toxicants, but there are few studies in humans.

**Objectives:**

We investigated the relationship of prenatal and child OP urinary metabolite levels with children’s neurodevelopment.

**Methods:**

Participating children were from a longitudinal birth cohort of primarily Latino farm-worker families in California. We measured six nonspecific dialkylphosphate (DAP) metabolites in maternal and child urine as well as metabolites specific to malathion (MDA) and chlorpyrifos (TCPy) in maternal urine. We examined their association with children’s performance at 6 (*n* = 396), 12 (*n* = 395), and 24 (*n* = 372) months of age on the Bayley Scales of Infant Development [Mental Development (MDI) and Psychomotor Development (PDI) Indices] and mother’s report on the Child Behavior Checklist (CBCL) (*n* = 356).

**Results:**

Generally, pregnancy DAP levels were negatively associated with MDI, but child measures were positively associated. At 24 months of age, these associations reached statistical significance [per 10-fold increase in prenatal DAPs: β = −3.5 points; 95% confidence interval (CI), −6.6 to −0.5; child DAPs: β = 2.4 points; 95% CI, 0.5 to 4.2]. Neither prenatal nor child DAPs were associated with PDI or CBCL attention problems, but both prenatal and postnatal DAPs were associated with risk of pervasive developmental disorder [per 10-fold increase in prenatal DAPs: odds ratio (OR) = 2.3, *p* = 0.05; child DAPs OR = 1.7, *p* = 0.04]. MDA and TCPy were not associated with any outcome.

**Conclusions:**

We report adverse associations of prenatal DAPs with mental development and pervasive developmental problems at 24 months of age. Results should be interpreted with caution given the observed positive relationship with postnatal DAPs.

More than one billion pounds of pesticides are used annually in the United States, three-quarters of which are used in agriculture (Donaldson et al. 2002). Recent biological monitoring studies indicate that pesticide exposures are widespread in the U.S. population, including to pregnant women and children [Barr et al. 2004; Berkowitz et al. 2003; Bradman et al. 2005; Centers for Disease Control and Prevention (CDC) 2006; Fenske et al. 2000; Whyatt et al. 2003].

There is evidence that fetuses can be exposed to pesticides. Pesticides pass through the blood–brain barrier and placenta and have also been found in amniotic fluid (Bradman et al. 2003). In addition, relative to their body size, young children may receive greater exposure than adults, because they eat, drink, and breathe more per unit of body weight (National Research Council 1993). They are closer to the floor and surfaces where pesticides may settle, and have extensive hand-to-mouth contact (Zartarian et al. 2000).

Fetuses and young children may also be more susceptible to potential neurotoxic effects of pesticides, because their brains are developing rapidly (Rice and Barone 2000). Organophosphates (OPs) and carbamates break down the enzyme acetylcholinesterase (AChE), already lower during pregnancy (Evans et al. 1988), allowing acetylycholine to build up in the neuronal junction. AChE inhibition disrupts cell replication and differentiation, synap-togenesis, and axonogenesis (Bigbee et al. 2000; Lauder and Schambra 1999). It is likely that pesticides also act through noncholinergic mechanisms involving alterations in expression and function of nuclear transcription factors (Dam et al. 2003). Recent studies have shown that fetuses and young children have lower-than-adult levels of detoxifying enzymes (paraoxonase or chlorpyrifos-oxonase) that deactivate OPs (Furlong et al. 2006; Holland et al. 2006), suggesting that they may be more vulnerable to these exposures.

Numerous animal studies have demonstrated that *in utero* or early exposure to OP pesticides affect neurodevelopment (Eskenazi et al. 1999). Although acute poisonings can cause neurologic impairment in children (Zwiener and Ginsburg 1988), few studies have assessed neurodevelopment of young children after low-level OP pesticide exposure (Grandjean et al. 2006; Guillette et al. 1998; Rauh et al. 2006; Ruckart et al. 2004; Young et al. 2005), and even fewer have done so using biomarkers of exposure. In an ecologic study (Guillette et al. 1998), 33 Yaqui Mexican 4- to 5-year-olds living in a valley where OPs, organochlorines (OC), and other pesticides were used were compared with 17 children living where no pesticides were used. Exposed children showed lower performance in gross motor, eye–hand coordination, draw-a-person, and delayed recall. In a study of 2-to 12-year-olds living in Mississippi and Ohio where methyl parathion was illegally sprayed in homes, exposed children showed decreased selective attention, delayed verbal memory, poorer motor skills, and behavioral problems; yet the authors deemed the results inconclusive because of inconsistent findings across sites (Ruckart et al. 2004). A recent investigation in 72 6- to 9-year-old Ecuadoreans found that those whose mothers worked in floricul-ture during pregnancy scored lower on the Stanford-Binet Copying Test than unexposed children. Concurrent exposure to OPs, as assessed by the children’s urinary dialkyl phosphate (DAP) metabolite levels, was associated with increased reaction time, but not with other domains of neurobehavior (Grandjean et al. 2006). Recently, Rauh et al. (2006) examined the relationship of maternal blood levels of chlorpyrifos, a diethyl phosphate pesticide, during pregnancy and performance on the Bayley Scales of Infant Development of 254 inner-city minority children through 3 years of age. Three-year-olds with high prenatal chlorpyrifos showed significantly more delays in psychomo-tor and mental development, and mothers reported more attention problems and symptoms of pervasive developmental problems.

In the present study, we investigated neurodevelopment and behavior in a cohort of children from primarily Latino, farmworker families living in the Salinas Valley of California. We previously reported that maternal prenatal DAP urinary metabolite levels in this cohort were significantly higher than for the general U.S. population sampled in the National Health and Nutrition Examination Survey (NHANES) survey (Bradman et al. 2005; CDC 2006). Additionally, we found prenatal DAPs to be associated with shortened gestational duration (Eskenazi et al. 2004) and abnormal neonatal reflexes, but not other clusters, on the Brazelton Neonatal Behavioral Assessment Scale (Young et al. 2005). We now present findings on the relationship of maternal prenatal and child DAP levels with performance on the Bayley Scales through 2 years of age and with maternal reports of behavior on the Child Behavior Checklist (CBCL) at 2 years of age.

## Materials and Methods

### Participants and recruitment

The Center for the Health Assessment of Mothers and Children of Salinas (CHAMACOS) of the Center for Children’s Environmental Health Research at the University of California, Berkeley, sponsors a prospective cohort study of the association of pesticides and other environmental exposures on the health of pregnant women and their children living in the agricultural Salinas Valley, California.

Detailed methods for the CHAMACOS study have been described elsewhere (Eskenazi et al. 2004, 2006). Pregnant women were screened for eligibility between October 1999 and 2000. Eligible women were ≥ 18 years old, < 20 weeks gestation, Spanish- or English-speaking, eligible for Medi-Cal, receiving pre-natal care at one of six community clinics serving primarily farmworker families, and planning to deliver at Natividad Medical Center. All participating women gave written informed consent. The institutional review board at University of California, Berkeley, approved the study.

We followed 531 women to delivery of a liveborn, surviving neonate. We excluded from analyses children who lacked a neuro-developmental assessment (*n* = 71), who did not have prenatal and relevant concurrent DAP metabolites measured (*n* = 3), were not singletons (*n* = 8), or had a medical condition which could affect assessment (*n* = 3; Down syndrome, deafness, hydrocephalus). We excluded Bayley results if raw scores were too low for standardization (*n* = 3, 1, and 1 for 6-, 12-, and 24-month assessments, respectively), or if assessed by a psychometrician who had completed too few for statistical adjustment (*n* = 4 at 6 months). Bayley analyses included 396 infants at 6 months, 395 at 12 months, and 372 at 24 months. In a few cases, a child had a valid score for only the Bayley Mental Developmental Index (MDI) or Psychomotor Developmental Index (PDI) (Bayley 1993). A total of 356 mothers completed the CBCL at the 24-month visit.

### Maternal interviews and assessments

Women were interviewed twice during pregnancy (mean = 14.0 and 26.6 weeks gestation), shortly after delivery, and when children were 6, 12, and 24 months old. Interviews were conducted in Spanish or English by bilingual, bicultural interviewers. Mothers were administered the Peabody Picture Vocabulary Test (PPVT) (Dunn and Dunn 1981) to assess scholastic abilities at the 6-month visit, and the Center for Epidemiologic Studies Depression Scale (CES-D) (Radloff 1977) at the 12-month visit. The Infant-Toddler HOME (Home Observation for Measurement of the Environment) (Caldwell and Bradley 1984) instrument was completed at 6 and 12 months of age, and 32 of 45 items were completed at 24 months. Prenatal and delivery medical records were abstracted by a registered nurse.

### Neurodevelopmental and behavioral outcomes. Bayley Scales of Infant Development

The Bayley Scales of Infant Development, Second Edition (Bayley 1993) assess the developmental functioning of infants and young children. The MDI characterizes a variety of cognitive abilities, and the PDI characterizes large muscle and fine motor coordination. Both scales were administered in Spanish and/or English by psychometricians blind to exposure. Psychometricians were trained using standardized protocols and were supervised for quality assurance by a clinical neuropsychologist. Assessments were performed in a private room at the CHAMACOS research office or in a recreation vehicle (RV) modified to be a mobile testing facility. Children were assessed on average (mean ± SD) at 6.6 ± 1.1 months, 12.8 ± 1.6 months, and 24.6 ± 1.1 months. Each scale is standardized by age to mean = 100 and SD = 15. Scores > 1 SD below the mean (i.e., < 85) indicate possible developmental delay.

#### CBCL

The 99-item CBCL for Ages 1.5–5 (Achenbach and Rescorla 2000) was administered to mothers to assess 2-year-olds’ emotional/behavioral problems and competencies. The CBCL has been widely used in cross-cultural research and collects data on a range of behavior problems, yielding scores for several syndrome scales and five scales designed to be consistent with *Diagnostic and Statistical Manual of Mental Disorders* (DSM) diagnoses (American Psychiatric Association 2000). *A priori,* we chose three scales to examine in relation to DAPs based on the animal literature (Eskenazi et al. 1999): the Attention Problems syndrome scale, which includes such items as “can’t concentrate” and “can’t sit still”; the DSM-oriented Attention-Deficit/Hyperactivity Disorder (ADHD) scale, which additionally includes such items as “gets into everything”; and the DSM-oriented Pervasive Developmental Disorder (PDD) scale, which includes such items as “avoids eye contact,” “rocks head, body,” and “unresponsive to affection,” which are “rated as very consistent with Asperger’s Disorder and Autistic Disorder” (Achenbach and Rescorla 2000). A score considered of “clinical” significance is > 98th percentile of the national normative sample, and of “borderline” clinical significance is > 93rd percentile.

### Pesticide exposure measurement

We measured six nonspecific OP DAP metabolites in maternal and child urine: three dimethyl (DM) phosphate metabolites (dimethylphosphate, dimethylthiophosphate, dimethyldithiophos-phate); and three diethyl (DE) phosphate metabolites (diethylphosphate, diethylthio-phosphate, and diethyldithiophosphate) (Bradman et al. 2005). These six metabolites represent the by-products of approximately 80% of OPs used in the Salinas Valley. Maternal urine was also analyzed for metabo-lites specific to malathion [malathion dicar-boxylic acid (MDA)], and chlorpyrifos [3,5,6-trichloro-2-pyridinol (TCPy)] (Olsson et al. 2003). MDA levels were missing for 91 women because of analytical problems. Values below the limit of detection (LOD) were assigned a value of LOD/(√2). Urinary creatinine concentrations were determined using a commercially available diagnostic enzyme method (Vitros CREA slides; Ortho Clinical Diagnostics, Raritan, NJ).

Urine specimens were usually collected at the time of interview and were aliquoted and stored at −80°C until shipment on dry ice to the CDC (Atlanta, GA). DAPs metabolites were measured using gas chromatography-tandem mass spectrometry and quantified using isotope dilution calibration (Bravo et al. 2002). Details of urine collection, analysis and quality control procedures, including detection limits and use of blanks and spikes, are described elsewhere (Bradman et al. 2005).

Serum levels of dichlorodiphenyl-trichloroethane (DDT) and dichlorodiphenyl-dichloroethylene (DDE) (*p,p*′-DDT, *o,p*′-DDT, *p,p*′-DDE), β-hexachlorocyclohexane, hexa-chlorobenzene, and polychlorinated biphenyls (PCBs) were measured in 360 prenatal blood samples using gas chromatography-high resolution mass spectrometry methods (Barr et al. 2003). OC and PCB measures were lipid-adjusted. Total PCBs were derived from serum levels of congeners 138, 153, and 180 (Needham et al. 2005). Lead was measured in cord blood using graphite furnace atomic absorption spectrophotometry.

### Data analysis

Nonspecific DAP metabo-lites (nanomoles per liter) were summed and transformed to the log_10_ scale. We created “pregnancy” DEs, DMs, and total DAP values by averaging the two log-transformed pregnancy measures. The two pregnancy total DAP measurements were correlated (*r* = 0.14, *p* = 0.005) and did not significantly differ (paired *t*-test = −0.28, *p* = 0.78). For 29 women, only one DAP measurement was available. Prenatal and postnatal DAP measures were uncorre-lated, so we placed both exposures into a single model for each outcome. Coefficients were similar to those in models containing either prenatal or postnatal exposures alone. Because a large proportion of women had nonde-tectable levels of MDA and TCPy, we categorized levels into three groups for each metabolite: < LOD for both pregnancy measurements, and for those with at least one detectable level, subdivided below and above the median of the average pregnancy level.

To assess the relationship between metabo-lite levels and Bayley performance, we constructed separate multiple regression models for MDI and PDI at each of the three time points: 6, 12, and 24 months. We evaluated MDI and PDI continuously using linear regression. We included the same covariates in all Bayley models. Covariates were selected for these analyses if they were related to conditions of testing [i.e., psychometrician (*n* = 4), location (office or RV), exact age at assessment]; related to neurodevelopment in the literature and associated (*p* < 0.10) with most outcomes [i.e. sex, breast-feeding duration (months), HOME score (continuous), and household income]; or consistently related to neurodevel-opment in the literature even if not in our data [i.e., parity and maternal PPVT (continuous)]. We classified household income as above or below poverty by comparing total household income to the federal poverty threshold for a household of that size (U.S. Census Bureau 2000). In addition to the variables we included, we examined the potential confounding effects of several other variables suggested by the literature (i.e., maternal age, education, depressive symptoms, active/passive smoking exposure during pregnancy, regular alcohol use during pregnancy, marital status, father’s presence in home, housing density, maternal work status, ≥ 15 hours out-of-home childcare/week), but they did not markedly alter the observed associations. For simplicity, the same set of covariates was used for CBCL models with three exceptions: maternal depression, found to be important (*p* < 0.10), was added, and psychometrician and assessment location were dropped, because scores were based on maternal report. Covariates in final models were categorized as noted in Table 1, unless otherwise specified above. To preserve the size of the analytic population, each missing covariate value was imputed by randomly selecting a value from participants with non-missing values. Maternal depression had the largest percentage of values requiring imputation (5%). Of remaining covariates, between 0% and 1.8% of values were imputed.

In secondary analyses, we controlled for some factors potentially on the causal pathway (birth weight, gestational age, abnormal reflexes) and re-ran models excluding low birth weight and preterm infants. We also considered whether controlling for other suspected neuro-toxicants (i.e., PCBs, lead, and DDT) and other high-level exposures in our population (i.e., β-hexachlorocyclohexane and hexa-chlorobenzene) (Fenster et al. 2006) altered our results for DAPs in the subsample with both DAPs and the other chemical. Furthermore, we examined interactions between child DAPs and child sex and, because we previously observed an association with maternal DDT and 24-month MDI (Eskenazi et al. 2006), between maternal DAPs and DDT. Finally, we re-ran models using log-transformed creatinine-adjusted metabolites for comparative purposes.

In addition, we performed longitudinal data analyses [generalized estimating equations (GEE)] of the relationship between DAPs and Bayley scores, which produced similar findings. These GEE models included indicators for age at assessment and interaction terms for most independent variables and age. We obtained a minor increase in precision when the effects of some potential confounders were assumed constant over time. In addition, assuming a single effect of DAPs over time also produced small increases in precision of the relevant regression coefficient estimators, but again the gains were slight. Thus, we only present the results from the cross-sectional analyses for ease of understanding.

## Results

Women were primarily young and parous, and relatively few had completed high school (Table 1). Most mothers were born in Mexico and spoke Spanish (89%), and 23% had been in the United States ≤ 1 year at enrollment. Nearly all (92%) lived with the child’s father, and two-thirds of families lived below the federal poverty threshold (U.S. Census Bureau 2000). Most women (82%) lived with one or more farmworkers and 43% worked in agriculture themselves during pregnancy. Almost no mothers consumed alcohol regularly during pregnancy, and few smoked or lived with a smoker. Half had symptoms of depression 1 year postpartum. The average maternal PPVT score was in the low normal range, averaging 86 ± 21.

One-third of children were first-born (Table 1). Almost all were initially breast-fed (96.6%), half for ≥ 6 months, and 29% still breast-fed at 12 months of age. Few children lived in home environments considered to be of low quality in terms of stimulation and interaction (HOME scores < 26) at 6 (8%) and 12 (< 1%) months of age.

The geometric mean (GM) of pregnancy DAPs was 114.9 nmol/L (Table 2). Child levels increased with age, with total DAPs averaging 45.5, 59.5, and 70.9 nmol/L at 6, 12, and 24 months of age, respectively. Maternal DAPs were uncorrelated with child DAPs (*r* = 0.04 with each age, *p* = 0.40 to 0.47); The 12- and 24-month DAPs were correlated (*r* = 0.20, *p* < 0.001) but neither was correlated with 6-month DAPs. DMs were similarly correlated, but there was no correlation of DE measurements across ages. However, at any given age, the DEs and DMs were significantly correlated (*r* = 0.28–0.50, *p* < 0.001).

As shown in Table 2, < 40% of MDA levels were above the LOD; the median detectable level was around 1 μg/L. Most (91%) mothers had detectable serum TCPy, with a median level around 4 μg/L.

At 6, 12, and 24 months of age, respectively, Bayley PDI scores (mean ± SD) were 96.4 ± 10.6, 106.0 ± 12.6, and 97.5 ± 10.6, and MDI scores were 95.7 ± 7.0, 100.6 ± 8.9, and 85.9 ± 11.8 (Table 3). The proportion of MDI scores < 85 increased dramatically at 24 months with 50% < 85 relative to 3–4% on earlier assessments. On the CBCL, more children scored in the clinical range on the DSM-oriented pervasive developmental disorder scale (14.4%) than the national reference sample (≤ 3%) (binomial test *p* < 0.0001), although similar to the expected proportion of children scored in the clinical range on the attention problems syndrome (2.0%) and ADHD (3.3%) scales (Table 3).

Table 4 presents the change [and 95% confidence intervals (CIs)] in PDI and MDI scores associated with a 10-fold increase in metabolites, controlling for covariates. Results were similar in unadjusted models. We did not observe any statistically significant associations between metabolite levels and PDI at any age. We observed an overall pattern of negative associations between pregnancy metabolites and MDI, and positive associations between concurrent child metabolites and MDI, which were not statistically significant until the 24-month assessment. Specifically, for every 10-fold increase in pregnancy DAPs, we found a 3.5 point decrease in the 24-month-olds’ MDI (95% CI, −6.6 to −0.5; p = 0.02); however, for every 10-fold increase in the DAPs at 24 months, we observed a 2.4 point increase in MDI (95% CI, 0.5 to 4.2; p = 0.01). We attribute the associations with total DAPs largely to the DM metabolites, which indicated a similar pattern of results.

Table 5 summarizes multiple regression results for CBCL outcomes. Because of small numbers (Table 3), we used less conservative “borderline” cut points for attention-related outcomes. We did not observe any significant associations for these two scales with any DAPs measurement. The only covariate we found to be significantly related to risk of attention problems and ADHD was maternal depression: Mothers who reported depressive symptomatology 12 months postpartum had nearly 3-fold odds for reporting that their 2-year-old had attention problems and ADHD [odds ratio (OR) = 3.1; 95% CI, 1.3 to 7.2; *p* = 0.01; and OR = 2.7; 95% CI, 1.2 to 5.9; *p* = 0.01, respectively].

We did, however, observe that children with higher prenatal and postnatal total DAPs were at significantly higher risk of PDD, with an approximately 2-fold increase in risk for each 10-fold increase in metabolites (prenatal DAPs OR = 2.3; 95% CI, 1.0 to 5.2, *p* = 0.05; 24-month DAPs OR = 1.7; 95% CI, 1.0 to 2.9, *p* = 0.04) We observed similar statistically significant associations for prenatal DM and 24-month DE exposures (Table 5). The only other covariate associated with report of PDD was maternal PPVT, which was negatively associated with risk of PDD (for each point increase in PPVT, OR = 0.98; 95% CI, 0.96 to 0.99; *p* = 0.001).

Controlling for birth weight, gestational age, abnormal neonatal reflexes, or additional chemical exposures did not substantially change our results. Results did not differ by sex of the child for any of the analyses. In addition, although we previously observed an association with maternal DDT and 24-month MDI (Eskenazi et al. 2006), we did not observe an interaction between DDT and prenatal DAPs. Overall, adjusting metabolites by creatinine yielded results similar to primary analyses, with some coefficients becoming slightly stronger and some weaker. The observed negative association of prenatal metabolites and 24-month MDI diminished somewhat (total DAPS: β = −3.0; 95% CI, −6.1 to 0.1; *p* = 0.06; DMs: β = −3.1; 95% CI, −5.9 to −0.4; *p* = 0.03). Creatinine-adjustment of child metabolites also reduced the positive associations of total DAPS (β = 1.7; 95% CI, −0.3 to 3.7; *p* = 0.09) and DMs (β = 1.4; 95% CI, −0.5 to 3.3; *p* = 0.14) on MDI at 24 months. CBCL results changed little with creatinine adjustment.

No significant associations were observed between MDA or TCPy and any Bayley or CBCL outcomes (Table 6).

## Discussion

We have previously reported in this cohort a relationship between DAP metabolites during pregnancy and both shortened gestation and poorer neonatal reflexes. These associations were seen primarily with dimethyl phosphate metabolites. Results from the present investigation suggest that DAP metabolite levels during pregnancy, particularly from dimethyl phosphate pesticides, may be negatively associated at 24 months with mental development (MDI) on the Bayley Scales and an increase in risk of maternally reported PDD. We also observed a curious positive association of postnatal DAPs with MDI; however, these postnatal metabo-lites were associated with an increased risk of PDD. Results persisted after controlling for other chemical exposures and were independent of previously observed associations of shortened gestation and poorer reflexes.

Although we observed associations between neurodevelopment and DAPs, we saw no such associations with metabolites specific to malathion (MDA) or chlorpyrifos (TCPy). Malathion is one of several pesticides devolving to DMs, and chlorpyrifos is one of several pesticides devolving to DEs; MDA and TCPy are only moderately correlated with DMs and DEs, respectively (MDA and DMs: *r* = 0.2 to 0.3, *p* < 0.001; DE and TCPy: *r* = 0.1 to 0.2, *p* < 0.04). Thus, the observed associations with DAPs may be attributed to compounds other than just these two. For example, oxydemeton-methyl, for which we cannot measure a specific metabolite, is a widely used DM in the Salinas Valley that is several times more neurotoxic than malathion (Castorina et al. 2003).

Few previous studies have examined low-level exposure to OP pesticides and children’s neurodevelopment using exposure biomarkers. Direct comparison with existing studies is complicated by differences in the exposure scenario or measurements and neurodevelopmental assessment methods. For example, a recently published cross-sectional study from Ecuador lacked biomarker-based pregnancy exposures, but DAP metabolites in 6- to 9-year-olds were associated with increased reaction times (Grandjean et al. 2006). In comparison, our study was larger and longitudinal, allowing for two pregnancy and three early childhood DAP measurements, but we assessed different outcomes and in younger children. Grandjean et al. (2006) reported that their DAP levels were similar to levels for 6- to 11-year-old U.S. children participating in NHANES (CDC 2006). DAPs in our children were lower than those in NHANES, but our children were considerably younger than those sampled in the national study. We observed a rise in DAPs from 6 months to 2 years of age, which is likely to continue as the children interact more with their environment. As in our study, Rauh et al. (2006) used the Bayley Scales and CBCL to assess neurodevelopment in a cohort of children up to 3 years of age, but measured chlor-pyrifos, a DE pesticide, in pregnancy bloods. Rauh et al. found inverse associations of chlor-pyrifos with mental development and pervasive developmental disorder, results that we found with total DAP and DM, but not DE or TCPy, metabolites. Unlike that study, we did not observe associations with PDI or attentional deficits. However, the exposure scenario in an agricultural community is likely quite different from that in an urban environment. Additionally, Rauh et al. (2006) found no effect at 2 years of age, only at 3 years.

We observed that DAPs in children were positively associated with neurodevelopment. We have no ready explanation for this finding. One possible explanation is that children with higher cognitive functioning may be more interactive with their environment, leading to higher exposure to pesticide residues. The pre-natal measurements do not suffer from the same issues of unknown temporal order in that they clearly preceded the measures of child development. Another possibility is that children who eat more fruits and vegetables may have higher DAP levels, but because of better diets have higher functioning.

DAP metabolites are limited as biomarkers of exposure. Recent studies suggest that urinary metabolite levels may reflect exposure not only to OP parent compounds, but also to the potentially less toxic ambient metabolites (Lu et al. 2005). Also, because exposure to OPs varies considerably from day to day, DAPs from spot urine samples may not represent average exposure over time. Although we observed significant correlations between some DAP measures over time, these correlations were weak. Preliminary results from our Center indicate that children’s DAP measurements more than a few days apart are uncorrelated, suggesting considerable intraperson variability (A. Bradman, personal communication). Any measurement of OPs in urine or blood reflect exposure during the brief (usually < 48 hr) antecedent period and therefore may not accurately reflect exposure throughout the entire critical period of neurodevelopment. While adjustment for creatinine muted the positive associations we observed with child metabo-lites and 24-month MDI, such an adjustment warrants caution. Although widely accepted in occupational studies of nonpregnant adults, creatinine adjustment may not be appropriate for metabolite levels in populations undergoing rapid physiologic changes, such as pregnant women and young children, due to high intraindividual variability in creatinine excretion (Boeniger et al. 1993).

Another limitation of this study is that we conducted many analyses and did not adjust for multiple comparisons. However, because of the interrelationships among the exposures and among the outcomes, the comparisons we made were not entirely independent. Specifically, the urinary DM and DE metabo-lites are subsets of the total DAPs and are therefore highly correlated with total DAPs at each time (DAPs and DMs: *r* = 0.9 to 0.96. *p* < 0.001; DAPs and DEs: *r* = 0.6 to 0.7. *p* < 0.001). In addition, MDI and PDI were moderately correlated at each of the three time points (*r* = 0.3–0.5; *p* < 0.001) and, although not as strongly, across ages (*r* = 0.1–0.2; *p* < 0.01), and the three CBCL outcomes we considered were associated with one another (chi-square *p* < 0.001 for all combinations). Thus, a Bonferroni correction might be considered unduly conservative (Bland and Altman 1995).

This study also has a number of strengths. Because children, especially those from economically impoverished environments, may be exposed to other environmental agents, we collected extensive information about additional environmental exposures and measured many with biomarkers, including lead, OC pesticides, and PCBs. We previously found that the CHAMACOS mothers had high serum levels of certain OCs, such as DDT and DDE, because many women had originated from Mexico where these pesticides were used more recently (Bradman et al. 2006). We have determined in our population that both the previously reported association of maternal DDT with MDI (Eskenazi et al. 2006) and the present association of maternal DAPs with MDI persisted when these exposures were modeled simultaneously.

Because our population was also relatively homogeneous, we could eliminate the potential for other covariates to explain the observed associations. Yet because the CHAMACOS population consisted mostly of children from low-income families, they were already at risk for poorer neurodevelopment (Black et al. 2000). Although we observed < 5% with MDI scores < 85 at 6 and 12 months of age, at 2 years of age almost 50% showed possible deficits. Although this drop in scores at 24 months might be attributed to a lack of developmentally stimulating environments, it is also possible that the Bayley Scales, although having internal validity in our population, may not be a clinically valid tool in Spanish or in Latino immigrant communities. Similar findings of drops in MDI scores at 24 months of age have been seen in a cross-sectional study of children in semiurban Mexico (Fernald et al. 2006) and in African-American and Dominican children in New York City (Rauh et al. 2006). The proportion of children with delays may decrease as they are exposed to more enriched environments such as preschool.

We also observed a very high proportion of CHAMACOS children with PDD. Although DSM-oriented scales based on maternal report are not directly equivalent to a DSM diagnosis (Achenbach and Rescorla 2000), this high report of PDD merits further investigation and follow-up, especially in light of a recent study that hypothesized that OP exposure during critical prenatal periods of neuronal migration may precipitate autism in a genetically vulnerable population (D’Amelio et al. 2005). In a case–control study of autism, D’Amelio et al. (2005) reported a significant association among Caucasian Americans between autism and certain variants of the paraoxonase gene (*PON1*) that encodes the enzyme responsible for OP detoxification. Thus, in future investigations, we will examine the interaction between OP exposure and *PON1* gene variants and enzyme expression on the observed associations with PDD (Furlong et al. 2006; Holland et al. 2006). Follow-up studies of this cohort as they enter school will determine the clinical significance and persistence of the observed deficits and may shed light on the positive relationships with postnatal exposure measurements.

In summary, we report an adverse association of prenatal organophosphate pesticide exposure as measured by DAPs with mental development and pervasive developmental problems at 24 months of age. This study is one of the first to examine the associations of both prenatal and postnatal organophosphate exposure on early neurodevelopment. The negative associations of pregnancy DAP measures with mental development should be interpreted with caution given the positive postna-tal relationships we observed and should be replicated in other populations.

## Figures and Tables

**Table 1:** Demographic characteristics (*n* = 447) of CHAMACOS study population, Salinas Valley, California, 2000–2001 [no. (%)].

Characteristic	Baseline	6 months	12 months	24 months
Child sex				
Female	226 (50.6)			
Male	221 (49.4)			
Maternal age (years)				
18–24	199 (44.5)			
25–29	139 (31.1)			
30–34	73 (16.3)			
≥ 35	36 (8.1)			
Parity				
0	144 (32.2)			
≥ 1	303 (67.8)			
Maternal education				
< 6th grade	197 (44.1)			
7–12th grade	163 (36.5)			
Completed high school	87 (19.5)			
Maternal country of birth				
Mexico	380 (85.0)			
United States	59 (13.2)			
Other	8 (1.8)			
Married or living as married				
Yes	366 (81.9)			
No	81 (18.1)			
Alcohol use during pregnancy				
Yes	4 (0.9)			
No	423 (99.1)			
Smoking during pregnancy				
Yes	23 (5.2)			
Lived with smoker	37 (8.3)			
No	387 (86.6)			
Maternal depressive symptioms (CES-D ≥ 16)				
Yes			203 (50.9)	
No			196 (49.1)	
Breast-feeding at time of assessment^a^				
Yes		208 (51.5)	124 (30.6)	30 (7.9)
No		196 (48.5)	281 (69.4)	350 (92.1)
Household income above poverty threshold^a^				
Yes	161 (38.0)	115 (28.8)	125 (33.8)	144 (40.9)
No	263 (62.0)	284 (71.2)	245 (66.2)	208 (59.1)
HOME score (points)^a^,^b^				
0–25		34 (7.6)	1 (0.2)	
26–36		360 (80.5)	232 (55.9)	
> 36		53 (11.9)	182 (43.9)	

Percentages represent percent of known values.

a Values at 6, 12, and 24 months of age are limited to population that had a Bayley assessment performed at that time.

b HOME score at 24 months of age is not complete.

**Table 2:** Urinary DAP metabolite measurements in mothers and children and pesticide-specific metabolites (MDA and TCPy) in mothers (CHAMACOS Study, Salinas Valley, CA, 2000–2003).

		DAPs (nmol/L)^a^,^b^			Pesticide-specific metabolites (μg/L)^a^			
	Total DAPs	Total DMs	Total DEs		MDA^c^		TCPy^c^	
	No.	GM (95% CI)	GM (95% CI)	GM (95% CI)	Detect^d^	Median^e^	Detect^d^	Median^e^
Maternal								
1st pregnancy	442	113.5 (100.5–128.2)	83.7 (73.2–95.7)	16.1 (14.6–17.8)	33%	1.03	71%	3.76
2nd pregnancy	419	116.9 (106.2–128.7)	78.4 (70.3–87.6)	20.9 (18.6–23.5)	25%	0.96	82%	4.60
Average^f^	445	114.9 (105.7–125.0)	81.5 (74.3–89.5)	18.1 (16.7–19.7)	39%^g^	0.82^g^	91%^g^	3.54^g^
Child								
6 months	405	45.5 (39.6–52.3)	23.8 (20.4–27.8)	10.6 (8.9–11.9)	—		—	
12 months	394	59.5 (51.7–68.5)	32.9 (27.8–38.9)	15.2 (13.5–17.2)	—		—	
24 months	373	70.9 (61.4–81.9)	48.6 (41.8–56.6)	10.5 (8.8–12.6)	—		—	

CI, confidence interval.

a Urinary metabolites, not adjusted for creatinine.

b Measurements below the LOD were assigned a value of LOD/√2.

c *n* = 356 for pesticide-specific metabolites.

d Percent above the LOD; for average pregnancy, this represents percentage with at least one of two measures above the limit of detection.

e Median values for those with at least one measurement above the LOD.

f Average of baseline and 26-week maternal pregnancy measures.

g Classified as below the LOD if both pregnancy values are below the LOD. If one value is below the LOD, a value of LOD/√2 is substituted for below the LOD value before averaging.

**Table 3:** Neurodevelopmental scale measures at 6, 12, and 24 months of age and neurobehavioral outcomes at 24 months of age (CHAMACOS Study, Salinas Valley, CA, 2000–2003).

Developmental/behavioral assessment	No.	Mean ± SD	No. (%) with problem^a^
Bayley Scales of Infant Development			
MDI			
6 months	395	95.7 ± 7.0	17 (4.3)
12 months	393	100.6 ± 8.9	13 (3.3)
24 months	369	85.9 ± 11.8	184 (49.9)
PDI			
6 months	396	96.4 ± 10.6	51 (12.9)
12 months	392	106.0 ± 12.6	12 (3.1)
24 months	371	97.5 ± 10.6	56 (15.1)
CBCL			
Empirically based scales			
Attention problems syndrome			
Clinical (> 97th percentile)	356	—	7 (2.0)
Borderline^b^ (> 93rd percentile)	356	—	30 (8.4)
DSM-oriented scales			
ADHD			
Clinical (> 97th percentile)	356	—	10 (2.8)
Borderline^b^ (> 93rd percentile)	356	—	34 (9.6)
PDD			
Clinical^b^ (> 97th percentile)	355	—	51 (14.4)
Borderline (> 93rd percentile)	355	—	105 (29.6)

a For Bayley scales, “problem” is defined as scoring more than one standard deviation below the mean, i.e., < 85.

b CBCL category used for regression models. “Clinical” is a subset of “borderline.”

**Table 4:** Adjusted^a^ coefficients (β) (95% CIs) in points on the PDI and MDI of the Bayley Scales of Infant Development for a log_10_ unit increase in pesticide urinary metabolites.

Bayley Scales of Infant Development	MDI^a^	PDI^a^
Total DAPs		
6 months		
Prenatal	−1.15 (−2.89 to 0.59)	−0.71 (−3.28 to 1.86)
Child	−0.17 (−1.23 to 0.90)	0.39 (−1.18 to 1.97)
12 months		
Prenatal	−1.34 (−3.59 to 0.92)	−0.60 (−3.77 to 2.57)
Child	1.36 (−0.05 to 2.78)*	1.22 (−0.78 to 3.21)
24 months		
Prenatal	−3.54 (−6.59 to −0.49)**	−1.28 (−4.01 to 1.46)
Child	2.37 (0.50 to 4.24)#	1.06 (−0.62 to 2.74)
DMs		
6 months		
Prenatal	−0.95 (−2.52 to 0.62)	−0.55 (−2.88 to 1.77)
Child	−0.31 (−1.28 to 0.67)	0.28 (−1.17 to 1.72)
12 months		
Prenatal	−1.06 (−3.12 to 0.99)	−1.15 (−4.03 to 1.74)
Child	0.75 (−0.44 to 1.93)	0.46 (−1.22 to 2.13)
24 months		
Prenatal	−3.64 (−6.36 to −0.91)#	−1.24 (−3.70 to 1.21)
Child	2.01 (0.24 to 3.78)**	1.01 (−0.58 to 2.60)
DEs		
6 months		
Prenatal	−0.16 (−1.96 to 1.65)	0.02 (−2.63 to 2.67)
Child	0.24 (−0.78 to 1.25)	0.60 (−0.89 to 2.09)
12 months		
Prenatal	−1.14 (−3.51 to 1.22)	0.30 (−3.03 to 3.63)
Child	1.89 (0.21 to 3.58)**	1.91 (−0.46 to 4.27)
24 months		
Prenatal	−0.85 (−3.98 to 2.27)	−0.86 (−3.64 to 1.92)
Child	1.02 (−0.52 to 2.57)	0.30 (−1.07 to 1.67)

a Models adjusted for psychometrician, location, exact age at assessment, sex, breast-feeding duration, HOME score, household income above poverty threshold, parity, and maternal PPVT.

* *p* ≤ 0.10;

** *p* ≤ 0.05;

# *p* ≤ 0.01.

**Table 5:** Adjusted^a^ ORs (95% CIs) for syndrome scores in the clinicial (CL) or borderline clinical (BL) range on the CBCL at 24 months of age for DAPs urinary metabolites.

CBCL	Attention (BL)^a^	ADHD (BL)^a^	PDD (CL)^a^
Total DAPs			
Prenatal	0.77 (0.27–2.24)	1.34 (0.50–3.59)	2.25 (0.99–5.16)**
Child	1.41 (0.75–2.64)	1.11 (0.61–2.03)	1.71 (1.02–2.87)**
DMs			
Prenatal	0.78 (0.31–1.96)	1.27 (0.53–3.04)	2.19 (1.05–4.58)**
Child	1.54 (0.85–2.76)	1.10 (0.63–1.94)	1.52 (0.94–2.45)*
DEs			
Prenatal	0.78 (0.26–2.31)	0.59 (0.21–1.68)	0.88 (0.37–2.07)
Child	1.02 (0.61–1.71)	1.18 (0.72–1.94)	1.72 (1.12–2.64)**

a Models adjusted for sex, exact age at assessment, breast-feeding duration, HOME score, household income above poverty threshold, parity, maternal PPVT, and maternal depression.

* *p* ≤ 0.10;

** *p* ≤ 0.05.

**Table 6:** Adjusted^a^ coefficients (β) (95% CIs) in points on the MDI and PDI of the Bayley Scales for prenatal urinary metabolites specific to malathion (MDA) and chlorpyrifos (TCPy).

Bayley Scales of Infant Development	MDI^a^	PDI^a^
MDA		
6 months		
< LOD (reference)	—	—
< Median detected	0.98 (−0.85 to 2.81)	0.42 (−2.34 to 3.18)
≥ Median detected	−0.25 (−2.10 to 1.60)	−1.45 (−4.21 to 1.32)
12 months		
< LOD (reference)	—	—
< Median detected	0.95 (−1.55 to 3.46)	−0.53 (−4.05 to 3.00)
≥ Median detected	2.40 (−0.13 to 4.94)	0.75 (−2.81 to 4.31)
24 months		
< LOD (reference)	—	—
< Median detected	−1.09 (−4.51 to 2.32)	−0.73 (−3.87 to 2.41)
≥ Median detected	0.24 (−3.03 to 3.52)	0.33 (−2.68 to 3.35)
TCPy		
6 months		
< LOD (reference)	—	—
< Median detected	0.24 (−2.12 to 2.60)	−0.56 (−4.03 to 2.91)
≥ Median detected	0.08 (−2.29 to 2.44)	−0.21 (−3.69 to 3.27)
12 months		
< LOD (reference)	—	—
< Median detected	−0.45 (−3.67 to 2.76)	−0.70 (−5.26 to 3.86)
≥ Median detected	−0.65 (−3.88 to 2.58)	−1.62 (−6.20 to 2.96)
24 months		
< LOD (reference)	—	—
< Median detected	−1.02 (−5.34 to 3.31)	−2.65 (−6.50 to 1.21)
≥ Median detected	−1.94 (−6.26 to 2.37)	−2.72 (−6.57 to 1.12)

a Models adjusted for psychometrician, location, exact age at assessment, sex, breast-feeding duration, HOME score, household income above poverty threshold, parity, and maternal PPVT.
